# Curved Layer Fused Filament Fabrication Using Automated Toolpath Generation

**DOI:** 10.1089/3dp.2016.0033

**Published:** 2016-12-01

**Authors:** Thomas Llewellyn-Jones, Robert Allen, Richard Trask

**Affiliations:** ^1^ Advanced Composites Centre for Innovation and Science (ACCIS), Department of Aerospace Engineering, University of Bristol, Queens School, Bristol, United Kingdom.; ^2^ Department of Mechanical Engineering, University of Bath, Bath, United Kingdom.

**Keywords:** 3D printing, additive manufacturing, software, multimaterial printing

## Abstract

An automated method for the generation of curved layer toolpaths is demonstrated to produce 3D printed components with improved aesthetic and structural properties using fused filament fabrication printing. Three case studies are shown, which demonstrate the ability of the G-code generating algorithm to resolve concave and convex structures. The combination of conventionally printed layers and curved layers within a single print is also demonstrated by producing double skin curved layer sandwich structures with static *z* printed cores. Clear improvements in the surface finish of printed components using curved layer fused filament fabrication are shown visually.

## Introduction

Additive layer manufacturing (ALM) is a term describing a variety of methods for constructing components from three-dimensional (3D) model data.^1^ With the recent expiry of a number of key patents, the cost of many ALM techniques has reduced dramatically. As a result, these techniques vary from low-cost desktop consumer products to high-performance commercial manufacturing and prototyping. ALM techniques provide a number of distinct advantages over subtractive manufacturing methods in that they produce little or no waste, can build components more quickly, and can resolve internal features more easily.

ALM builds components layer wise, typically by dividing 3D model data into slices of equal thickness in horizontal planes. Each of these layers is then produced consecutively, with the technique of feedstock consolidation varying significantly between different forms of ALM. A number of ALM methods (such as stereolithography [SLA] and selective laser sintering [SLS]) make use of a bed of feedstock material on the build platform, which is solidified in selective regions through one of a number of methods. SLA printing requires a photocurable resin, which is then exposed to laser light to cross-link the polymer, while SLS printing also makes use of a laser to selectively sinter regions of material into a solid structure.^1^ As many of these methods rely on precisely calibrated optical systems to solidify the feedstock material, they exhibit some of the highest resolutions and lowest defect rates available with ALM. Consequently, they typically have high associated machine and material costs, resulting in these methods being used almost exclusively for high value commercial applications. Despite this, many high performance materials have been developed to be compatible with these methods.^2,3^

One of the most common forms of ALM is fused filament fabrication (FFF), in which thermoplastic filament is fed to a moving print head, which consists of a heated metal block above a fine nozzle. The thermoplastic feedstock is melted within the heated block and pressure from additional filament being driven to the head forces the melted plastic through the print nozzle. The print head moves along a precise toolpath, while polymer is extruded and deposited on the print bed to construct the desired component. As a result of its relative simplicity, FFF is inexpensive compared with other ALM techniques. Consequently, there is a wide range of materials available for FFF printing, and multimaterial printing is becoming increasingly reliable.^4,5^

For deposition-based ALM methods, which utilize a moving print head such as FFF, toolpathing becomes an important factor in determining the quality of the finished component, both mechanically and aesthetically.^6^ As aforementioned, ALM methods typically slice topological model data horizontally and print layers with a constant *z-*value consecutively. There are a number of disadvantages to producing components with static *z*-values within a single layer. The mechanical failure of printed components is often caused by failure between two layers within the model.^7^ With static *z*-value layers, the position of these interlayer defects is often determined by the easiest orientation for printing, and may not be optimal for the mechanical properties of the component. Using dynamic *z*-values and producing curved layers, a component can be printed along its skin, thereby improving its mechanical properties and surface finish.^8^ This combination of toolpathing and printing is known as curved layer fused filament fabrication (CLFFF), and there are a number of examples of methods to generate these toolpaths.^9–11^

As a result of the traditional static *z*-value layers used in FFF, most printer designs have matching *x* and *y* axes in terms of speed and acceleration, while the *z* axis is significantly slower. This is because the *x* and *y* axes are belt driven and manipulate the print head, whereas the *z* axis moves the entire build platform through a wide pitch leadscrew.^12,13^ Due to the independence and orthogonality of each axis, these are generally called Cartesian style printers. This is not an issue in traditional FFF as the distances moved in the *z* direction are small (0.1–0.4 mm) and only occur at the end of each layer. This is, however, a significant hindrance in CLFFF, as print lines move dynamically in the *z* direction and so print speed is limited to the maximum speed in the *z* axis, which is dramatically slower than what the *x* and *y* axes can achieve. To overcome this issue, this study makes use of a delta style parallel robot, where all three axes are matched, with the print head manipulated by three arm pairs working in parallel. Print head speeds of 300 mm/s are achievable in any direction, compared to a limit of ∼5 mm/s in the *z* axis on a typical Cartesian style printer. This work demonstrates the use of a novel algorithm for producing CLFFF toolpaths to construct arbitrary 3D model files, with the ability to produce scaffold material, as well as dual skin models with a functional core structure.

## Materials and Equipments

### Delta-style FFF printer

**FIG. 1.**
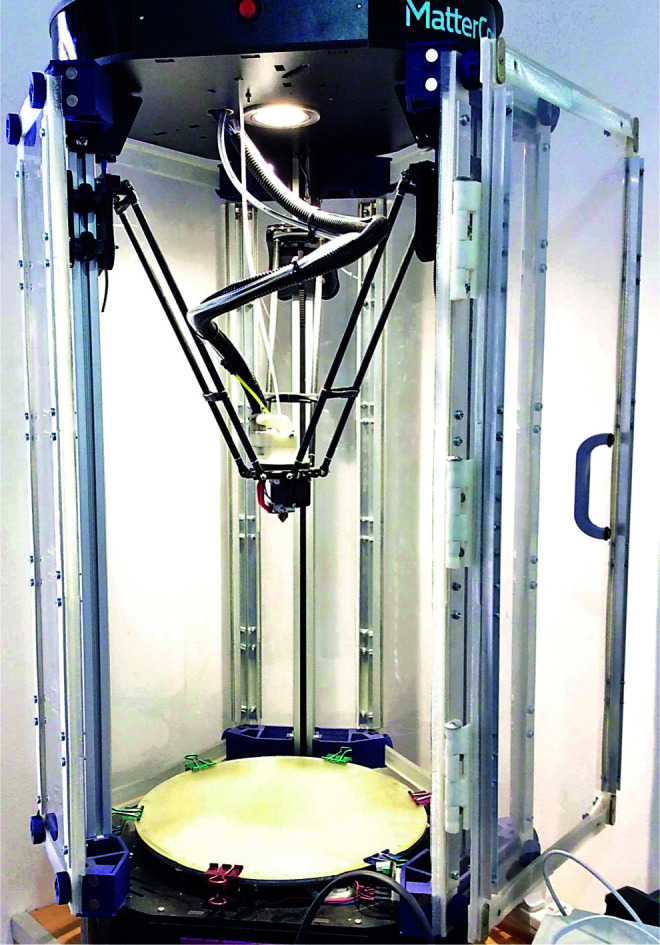
Modified Rostock Max v2 fused filament fabrication system used in this study.

To implement curved layer toolpaths within FFF, a delta style Rostock Max v2^14^ parallel robot was used. Parallel robots operate by manipulating a number of arms to support and maneuver an end effector plate, with the arms working in parallel to manipulate the base plate, as opposed to more traditional serial robots where each effector is independent and unconstrained by the other effectors.^15^ This parallel working method has the advantage of increased rigidity, as the error in position of the effector plate is averaged across the error in each axis, rather than cumulative. This rigidity is particularly useful in performing consecutive prints where the head must return to a known position to deposit further layers on top of previously printed material, such as printing a model on top of a scaffold structure after changing the material feedstock. The delta style parallel robot also has the advantage that all three axes are effected by the same arms and motors, meaning accelerations for all three axes are the same. Typically, Cartesian printers utilize a leadscrew-driven *z* axis, which reduces the potential acceleration of this axis and limits print speeds for CLFFF. The printer used in this study has been retro fitted with a number of upgrades to facilitate the CLFFF process used in this case. Significantly, 32-Bit hardware^16^ is used in tandem with the latest dc42 fork of the RepRapFirmware^17^ and auto calibration methods^18^ to aid in processing the additional complexity of CLFFF toolpaths at typical FFF manufacturing speeds. In addition, an E3D v6 hotend and Flex3Drive extrusion system have been installed below the effector plate to relieve geometric constraints due to the standard print heads geometry and to assist in the extrusion of Thermoplastic Elastomer (TPE) feedstocks. The modified Rostock Max used in this study is pictured in Figure 1.

## Slicing Algorithm

A slicing algorithm has been developed to simplify the process of producing curved layer toolpaths for arbitrarily shaped components. To maintain simplicity, the algorithm has been designed to receive model data from an stereolithography (STL) file, in the same manner as the majority of slicing programs. The script used produces separate G-code files for each section of the print (e.g., scaffold, buffer layers, and model skins) to allow the feedstock material to be changed for each section of the model. This is essential when using insoluble scaffold material, as a flexible buffer layer must then be printed between the scaffold and model to allow for separation of each section. Currently the slicing algorithm requires the input model file to be a thin skin, with thickness much less than a single layer thickness. This skin is then repeated over a number of layers to produce a part with the desired thickness, and as such it is only possible to produce extruded 2D shapes with varying raster angle in each layer. The script is written in MATLAB.^19^

### Scaffold structure

The first G-code file generated by the algorithm produces a scaffold structure upon which the curved layer model will be printed. The scaffold is defined by a set of user input vectors, which are built up to match the bottom surface of the model and act as a temporary toolplate. The use of a vector system allows for any repeating cellular structure to be produced. The scaffold structure is printed with conventional static *z* layers as the resulting structural weaknesses are not of concern. The method to produce the scaffold works by producing a grid extending beyond the part and then clipping the grid lines to the outline of a top down projection of the model.

To clip the scaffold grid lines to the required print area, the outline of the shape must be determined. Initially the model data are loaded from an STL file, which contains a list of vertices and facet normals, which produce a triangular tessellation of the model. The model data are then centered in the *x* and *y* axes and translated in the *z* direction so that its lowest value for *z* matches the height of the print bed. This allows the coordinates for the model in computer aided design (CAD) space to be used for the printing coordinates, as this corresponds to the center of the Delta robot build platform, with the model just touching the platform surface. Depending upon user input, the model is then shifted above the build platform, to allow for additional scaffold material underneath the entire model, which produces a better surface finish on the bottom surface of the printed part.

The *x* and *y* coordinates of the outline are determined by producing a binary image of a top down projection of the model and using a Moore-Neighbor tracing algorithm to determine the outline of the shape exterior, as well as any internal holes. The outlines of each feature are stored separately, and points are sorted in a clockwise direction from the part center. This outline shape is then extended by a given distance using a preexisting MATLAB polygon manipulation library,^20^ and the convex hull of the resulting polygon is found to produce the effective scaffolding area. While this can result in excessive print area by ignoring concave features in the projection outline, it significantly reduces travel moves and simplifies the structure of the scaffold.

The coordinates at which each grid line intersects the model outline are stored, as these are the start and end points for each print line in the scaffold. Each of these is a print move, whereas the movement from the end point of one print line to the start of the next is a travel move. When all the print lines in the layer are calculated, a nearest-neighbor approach is used to determine the order of the print lines in the layer, to reduce print times and minimize long travel moves. The *x* and *y* coordinates of a large number of reference points are then taken along each print line, and the *z*-value for the bottom layer of the model at each of these coordinates is calculated by interpolation over the model surface.

As aforementioned, if selected by the user the model file is shifted off the print bed by several millimeters to include space for scaffolding and buffer layers under the entire part, so the initial layers of the scaffold ignore changes to any of the initially calculated print lines. Consecutive static *z* layers are then included in the print file with the *z*-value increasing by the user input layer thickness, until the *z*-value of the current layer is equal to or greater than the *z*-value two layers below the bottom layer of the model at any of the reference points along any print line. It is then necessary to modify any effected print lines, either by retracting the start and end points of the print line further toward the interior of the model or splitting the print line into two or more separate print lines, depending upon the type of intersection. The modification required is easily found by determining whether the point(s) along the print line, which are above the model *z*-values, extend to the end of the print line. If this is the case, then the line is shortened until its entire length sits beneath the model. If this is not the case, the line is split at the two points of intersection. This process is iterated until no remaining reference points sit below the model.

Once this is completed, the coordinates of all print lines have been determined, and the next step is determining extrusion values. This is a volumetric calculation, requiring user inputs for the filament diameter and layer thickness. Using these, the extrusion value for a given print line is calculated by:

\begin{align*}
E = {w_t}{l_t} \sqrt {{{ \left( {{y_2} - {y_1}} \right) }^2} + {{ \left( {{x_2} - {x_1}} \right) }^2}}
\end{align*}

Where *E* is the extrusion value, *w_t_* is the wall thickness of each print line (determined by the nozzle diameter), *l_t_* is the user input layer thickness, and *x_1_*, *y_1_*, *x_2_*, *y_2_* correspond to the *x* and *y* coordinates of the start and end points, respectively, with all values in millimeters. It is assumed that the cross section of a printed line is roughly rectangular. The extrusion value corresponds to the length of filament which is driven through the extruder for a given print line. Once the extrusion values for all the print lines are calculated, the print file for the scaffold is written to file, using the user input values for print and travel speeds. At each travel move, extra G-code commands are inserted to quickly raise the print head and retract the filament several mm before travelling and return the head and filament after travelling. This helps to prevent filament stringing and dragging the print head across the scaffold.

### Curved layer model

Once the scaffold file has been completed, the algorithm then produces a buffer layer file (if selected by the user) and the model file. Both of these, along with the top layers of the scaffold, are generated using the same slicing process. Traditional slicing considers a 3D model and takes a slice of the model along a horizontal plane. To produce curved layer toolpaths, a slice must instead be taken in a vertical plane. To allow for maximum control, the slicing method was made so that any angle of this plane to the *x* axis, and therefore, the direction of each of the print lines in a layer, could be used. This also allows for different slicing angles between layers, resulting in user defined toolpath raster angles. Consequently, slicing time increases significantly, but print files can be designed to maximize part strength within specific 3D planes.

To produce a smooth finish on the outer edges of the part, an outline of the shape is printed at the beginning of each layer, which is subsequently infilled. This outline is the one originally determined during the scaffold slicing stage. As the outline for each layer is identical due to the previously described limitations of the slicer, it is only necessary to calculate these coordinates once, with each subsequent layer outline simply shifted in the *z* axis by a single layer thickness. The *z*-value at each point is interpolated from the model data using a linear scattered interpolation method, and these coordinates form the outline print for each layer.

Once the outline is completed, the slicing process for each individual layer begins. A vertical plane is generated at an angle (given by user input) to the *x* axis at one edge of the component. This plane is iteratively shifted by one wall thickness toward the far edge of the part, and any triangular elements, which are contained between two neighboring planes, are taken as being part of a single slice. As with the scaffold structure, the points of intersection between the print lines and the shape outline are noted as start and end points for a single print line, and the *x* and *y* coordinates of a large number of points along this print line are determined. The *z*-values for each point are interpolated from the model data as before. Unlike the scaffold material, where the *z*-values were used as limits for the scaffold height, these *z*-values are now used as the positions for the print lines, producing curved layers. Components with steep gradients will require large numbers of points along each print line to properly resolve the model shape, which can cause the print speed to slow significantly, as the motor controller automatically decelerates the print head toward the end of each print line. With too many small print lines in succession, the full speed of the printer is never reached, and therefore, steep gradients should be avoided where possible. As typical printers utilize an 8-bit processor, the buffer in the control board may also quickly saturate if large numbers of small print lines are used, resulting in jerky print head movement, which significantly affects the quality of the component. Extrusion values are calculated in the same manner as the scaffold structure print lines.

This process is iterated through all the unique user input layer angles. Each unique angle is only calculated once, as the layers must be identical as previously described. Once this is completed, the full sequence of layers is compiled, with each consecutive layer shifted by one layer thickness above the previous layer in the *z* direction. At this stage the compiled print data are written to a G-code file.

## Case Studies

To experimentally demonstrate the effectiveness of the CLFFF slicing algorithm, three case study example components were fabricated as follows.

### Case study 1: vehicle body panel

**FIG. 2.**
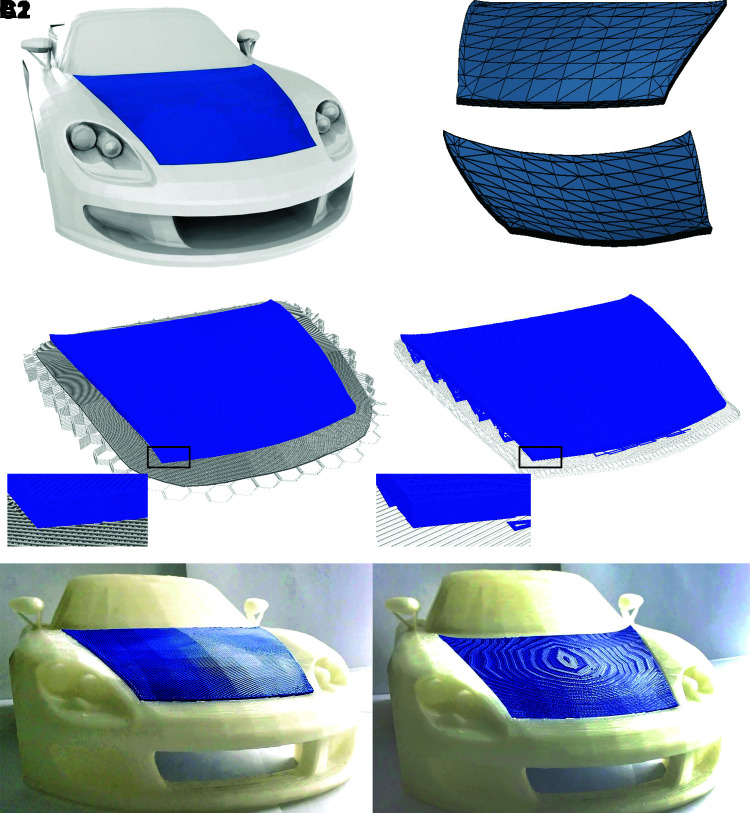
Vehicle body panel: **(A1)** CAD render of vehicle with selection highlighted in *blue*; **(A2)** Upper and lower surfaces of mesh for conventional slicing; **(B1)** Visualization of CLFFF toolpath generated for manufacturing; **(B2)** Visualization of conventional FFF toolpath for comparison; **(C1)** CLFFF manufactured part fitted to model; and **(C2)** Conventional FFF manufactured part fitted to model for comparison. CLFFF, curved layer fused filament fabrication; CAD, computer aided design; FFF, fused filament fabrication.

The first example demonstrated in this study is the manufacture of a scaled bonnet panel for a production vehicle, which measures ∼120 × 120 mm at its largest dimensions, adapted from an open source design.^21^ The desired surface region of the CAD data file to be manufactured is carefully selected using a suitable software package. In this study Autodesk Meshmixer^22^ was used, and the selected region of the mesh file is highlighted in Figure 2-A1. For this example, the slicing algorithm is used in its basic function to create a doubly curved convex surface panel with complex outer geometry and of precise exterior dimensions with uniform thickness. The CLFFF part is manufactured in a similar style to our previous work,^9^ consisting of a sacrificial scaffold structure manufactured from PLA using conventional toolpathing and a TPE^23^ buffer layer that follows the part surface geometry to support the CLFFF part during manufacturing. The panel consists of 15 individual CLFFF layers with consistently varying raster angles giving an overall panel thickness of 3.0 mm, and a detailed digital representation of the individual generated toolpaths is detailed in Figure 2-B1. The ability to vary raster angles ensures maximum and uniform part strength in the plane of the CLFFF surface. To further demonstrate the advantages of CLFFF toolpathing, an identical part was manufactured using Simplify3D, a commercial slicing software.^24^ This part uses the inbuilt support structure function to manufacture the complex geometries of this panel. The input mesh data are a 3.0 mm in-plane extrusion of the panel skin file imaged in Figure 2-A2, and the conventional generated toolpath is detailed in Figure 2-B2. In both examples the final parts were manufactured using colored PLA for clarity and then fitted to a physical model of the CAD file used for comparison and are imaged in Figure 2-C1 and C2, respectively.

### Case study 2: shoe insole

**FIG. 3.**
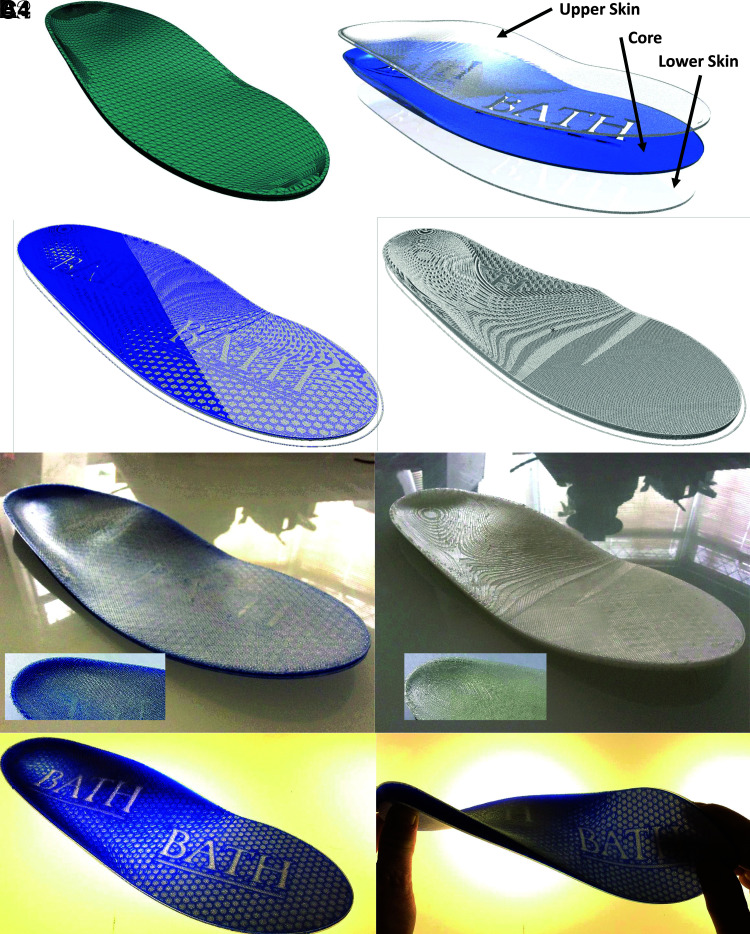
Shoe Insole: **(A1)** Original part mesh; **(A2)** Adapted part split into three regions; **(B1)** Visualization of CLFFF toolpath generated for manufacturing; **(B2)** Visualization of conventional FFF toolpath for comparison; **(C1)** CLFFF manufactured part, surface detail *inset*; **(C2)** Conventional FFF manufactured part for comparison surface detail *inset*; **(C3)** Backlit CLFFF manufactured part revealing core structure; and **(C4)** Backlit CLFFF manufactured part demonstrating its flexible properties.

The second example manufactured in this study is a modified insole for a UK size nine male shoe, measuring ∼265 × 90 × 20 mm.^25^ The basic mesh structure is detailed in Figure 3-A1. To demonstrate the ability to use CLFFF toolpaths in tandem with conventional toolpathing techniques, the model is divided into three separate regions as detailed in Figure 3-A2. In this case both core and lower skin structures are manufactured using toolpaths generated by Simplify3D software. This structure then acts as the support structure for the final form fitting CLFFF layers and negating the necessity for the scaffold and buffer layers used in case study 1. Figure 3-B1 illustrates a region of the CLFFF toolpath used in this study demonstrating how the conventional model supports the CLFFF layers, with raster angle being varied similarly to case study 1.

For comparison an identical part was manufactured by combining all three model parts again using Simplify3D. The generated conventional toolpath is visualized in Figure 3-B2, with the final portion of the code missing to reveal how both core and upper skin structures are constructed simultaneously in this case. To improve the function of the final parts, all components were manufacturing using TPE material,^23^ and final comparisons of CLFFF and conventional components are imaged in Figure 3-C1 and C2. The core structure of the CLFFF component was constructed using a colored TPE to allow better visual assessment of the quality of the manufacture process and further demonstrate additional benefits of CLFFF toolpathing. Figure 3-C3 is a backlit image of the same component revealing its detailed core structure in this case, while Figure 3-C4 demonstrates the performance of the CLFFF TPE insole under mechanical stress.

### Case study 3: dished sandwich panel

**FIG. 4.**
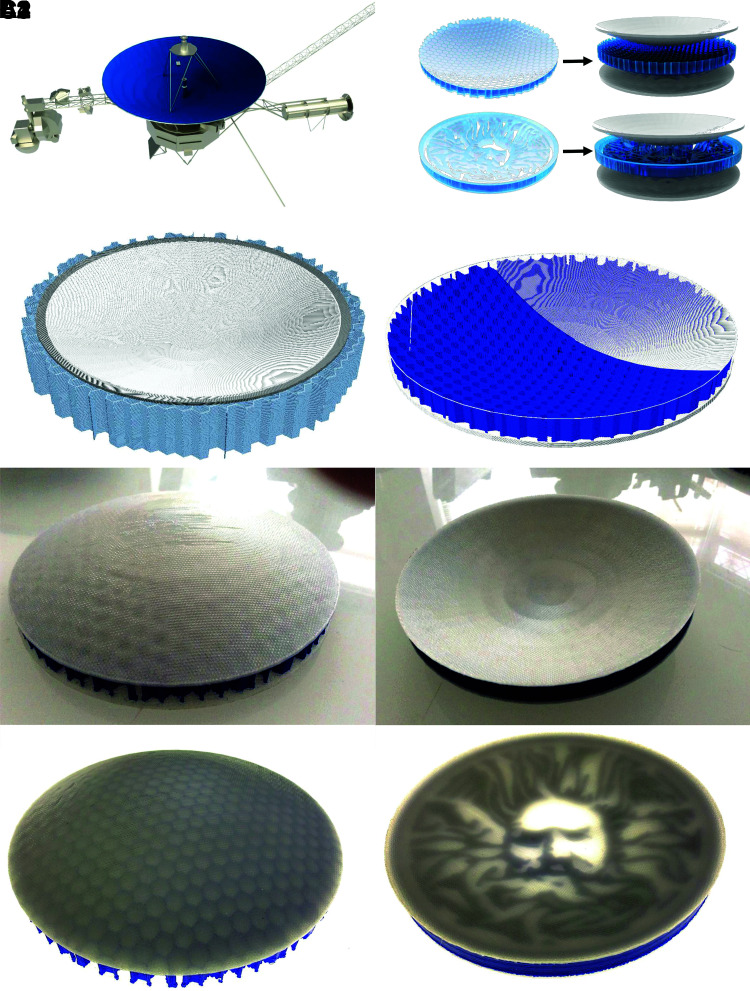
Parabolic dish sandwich panel: **(A1)** Original model with highlighted part mesh; **(A2)** Adapted sandwich panels split into three regions; **(B1)** Visualization of CLFFF toolpath generated for lower surface manufacturing; **(B2)** Visualization of CLFFF toolpath for upper surface manufacturing; **(C1)** Lower surface of honeycomb core sandwich panel; **(C2)** Upper surface of logo core sandwich panel; **(C3)** Lower surface of honeycomb core sandwich panel backlit revealing core structure; and **(C2)** Upper surface of logo core sandwich panel backlit revealing core structure.

The final case study demonstrated is a concave parabolic reflector adapted from the freely available NASA voyager space probe model^26^ detailed in Figure 4-A1. The parabolic dish model used in this case measures ∼150 mm in diameter with a depth of 22 mm and in-plane thickness of 14 mm. This example is used to show how CLFFF can be used to create multifunctional sandwich panel structures with complex doubly curved upper and lower surfaces and intricate core structures. Figure 4-A2 details two core structures investigated in this study. To manufacture these components, the lower part surface is constructed identically to the vehicle panel in case study 1, and example G-code is visualized in Figure 4-B1. The core structure is then manufactured using conventional toolpathing, allowing the inclusion of complex core structures. In this example a honeycomb structure and university logo are used, but in practice any desired function could be added to the core structure, as long as the core structure can sufficiently support the upper surface structure as detailed in Figure 4-B2. Furthermore, in this example parts are manufactured to have identical upper and lower surfaces for clarity, but the method can be used with differing upper and lower part surface geometries as detailed in case study 2, for example. Figure 4-C1 and C2 images the upper and lower surfaces of the CLFFF manufactured sandwich panel structures, respectively, and Figure 4-C3 and C4 reveals the core structures of the same parts using a backlight.

## Discussion and Conclusions

This study shows the ability to automatically generate curved layer G-code toolpaths for arbitrary shapes from a typical CAD model file, which are then successfully printed with a delta shape FFF 3D printer. Experimental results have clearly shown the efficacy of using curved layers to improve the surface finish of a printed component. Three case studies were used to demonstrate curved layer toolpath generation and printing of both concave and convex shapes. Comparisons of the print components and CAD files show high fidelity, with no observed distortion of the model during slicing.

At present, model files are limited to extruded 2D skins, and further work is required to implement this slicing procedure in full 3D models. It has also been shown that combining the use of curved layer and conventional static *z* layers is possible, which will enable simple inclusion of curved layer printing on the outer layers of conventionally printed parts to improve surface finish and reduce the risk of layer delamination. Combining these two slicing methods also minimizes the risk of print failure from curved layers with steep gradients and provides a route to printing full 3D objects with curved layers rather than extruded skins. Structural testing is required to determine the effect of this curved layer printing on the mechanical properties of final components.

The case study samples used in this study were chosen specifically to prove the effectiveness of the slicing algorithm and as such are not suitable for mechanical testing. Very long print times for these case studies also make production of a statistically significant sample size prohibitively slow. Mechanical testing of components from both traditional FFF and CLFFF, for direct comparison, is the subject of ongoing work. The ability to control the direction of print lines in a given layer will enable tailoring of structural properties by making use of the orthotropic properties of FFF printed materials, which may be enhanced by the use of short fiber reinforced thermoplastic filaments as a feedstock. Potential applications for this algorithm include the following: printing embedded electronics in components with complex geometries thereby forming a structural computer; printing of bespoke, personalized biological scaffolds for bone regrowth; and inclusion of smooth vacuoles within thin shell structures with complex topology for self-healing purposes.^27–29^
